# Brain Organoids and Assembloids—From Disease Modeling to Drug Discovery

**DOI:** 10.3390/cells14110842

**Published:** 2025-06-04

**Authors:** Aderonke O. Ajongbolo, Sigrid A. Langhans

**Affiliations:** 1 Division of Neurology, Nemours Children’s Health, Wilmington, DE 19803, USA; ronkeaj@udel.edu; 2 Biological Sciences Graduate Program, University of Delaware, Newark, DE 19716, USA

**Keywords:** brain, cerebellum, organoid, neurodevelopmental disease, neuropsychiatric disorder, neurodegeneration, substance abuse, brain cancer, high-throughput screening (HTS), drug discovery, cancer neuroscience

## Abstract

Brain organoids are self-organized, three-dimensional (3D) aggregates derived from human embryonic stem cells, induced pluripotent stem cells, or primary organs with cell types and cellular architectures resembling those of the developing human brain. Recent studies have shown the use of region-specific brain organoids for modeling various diseases ranging from neurodevelopmental and neurodegenerative diseases to different brain cancers, which have numerous applications in fundamental research and the development of new drugs, personalized treatment, and regenerative medicine. Consequently, the use of brain organoids in drug discovery is complex and challenging and still an emerging area in this field. This review article summarizes the primary stem cells used in brain organoid generation, region-specific brain organoids, and the functional assays used in their characterization. In addition, we discuss the use of brain organoids in modeling neurodevelopmental and neurodegenerative diseases and pediatric brain cancers, as well as the application of organoids, assembloids, and tumoroids in cancer neuroscience. We further explore the recent advances in using brain organoids in high-throughput screening to improve their use for drug discovery.

## 1. Introduction

Organoids refer to cells cultivated in a specific three-dimensional (3D) environment in vitro, forming mini clusters that autonomously organize and differentiate into functional cell types, mirroring the structure and function of an organ in vivo [1]. Brain organoids are generated from human pluripotent stem cells (hPSCs) that self-assemble into an organized structure featuring progenitor, neuronal, and glial cells, resembling the human brain [2,3,4]. Additionally, certain brain organoids may display structural characteristics that emulate specific brain regions, known as organoids of a particular region [5]. In contrast to traditional two-dimensional (2D) cell cultures, brain organoids aim to replicate the human brain not just at the cellular level but also regarding overall tissue structure and developmental pathways, creating a unique chance to study human brain development, function, and diseases that are often challenging to investigate through direct experimentation [2]. Throughout the years, brain organoids have been utilized to replicate numerous neurodevelopmental and neurodegenerative disorders, alongside various types of brain tumors, offering promise for the development of new medications. Nevertheless, the application of brain organoids as a model in high-throughput screening (HTS) remains a novel and expanding area of research. Progress in technologies related to hPSC-derived brain organoids for disease modeling will enhance our understanding of these models in HTS, thereby refining the drug discovery and development process that utilizes brain organoids. In this article, we review major stem cells used in the generation of brain organoids and provide an overview of assembloids. We discuss region-specific brain organoids and recent research findings in disease modeling and describe their potential use in drug screening and development.

## 2. Stem Cells

Stem cells are characterized as cells that can self-renew and differentiate into specialized functional cells [6,7]. Because of their ability to both self-replicate and develop into several types of cells, stem cells are crucial at various stages of development. During the initial stages of embryonic development, pluripotent stem cells serve as progenitors for all tissue types. In contrast, in later developmental stages, tissue-restricted stem cells generate cells with specific functions [8]. The primary features of stem cells include self-renewal (the ability to proliferate extensively), clonality (typically originating from a single cell), and potency (the capacity to develop into various cell types) [9]. Stem cells can be divided into four types based on their origin: embryonic, fetal, adult (which can be resident or tissue-specific), and induced pluripotent stem (iPS) cells. Both embryonic and iPS cells are pluripotent, fetal and perinatal stem cells are generally multipotent, and adult stem cells tend to be either oligopotent or unipotent [9,10]. Pluripotent stem cells can develop into various cell types originating from all three germ layers, the ectoderm, endoderm, and mesoderm, which give rise to all tissues and organs [11]. Multipotent stem cells are present in most tissues and can differentiate into cells from a single germ layer [12], and lastly, oligopotent stem cells can self-renew and create two or more lineages within a specific tissue. In contrast, unipotent stem cells can only transform into one specific cell type and form a single lineage [13,14]. Stem cells have applications in cellular therapies to replace damaged cells or regenerate tissues. Furthermore, research on stem cells has enhanced our comprehension of developmental processes and the origins of diseases [6,8,10]. The most used stem cells for generating brain organoids are embryonic and iPS cells.

### 2.1. Embryonic Stem Cells

The isolation of human embryonic stem cells (hESCs) in the late 1990s opened new avenues in human developmental biology and regenerative medicine [15]. Embryonic stem cells (ESCs) are obtained from embryos, as indicated by their name. Initially derived from mouse embryos, the 1998 extraction of ESCs from human embryos by James Thomson captured significant attention [16]. ESCs are pluripotent and emerge from the blastocyst’s inner cell mass, a stage of the pre-implantation embryo occurring 5–6 days after fertilization [17]. The blastocyst consists of two layers: the inner cell mass and the outer cell layer, consisting of cells known as trophoblasts. To derive embryonic stem cell (ESC) lines, cells from the inner cell mass are isolated from the trophoblasts and cultured in a controlled environment [18]. The identification of ESCs relies on the presence of specific transcription factors such as Nanog and Oct4 [19,20]. These factors are crucial in keeping the stem cells undifferentiated and enabling self-renewal. ESCs maintained in an undifferentiated condition without genetic abnormalities can be propagated as an ESC line. These cells can be cryopreserved and later thawed for additional cultures and experiments [21]. Proper culture conditions are essential for preserving ESCs in an unperturbed and undifferentiated form [22]. A feeder layer of mouse embryonic fibroblast cells (MEFCs) or a medium containing the anti-differentiation cytokine leukemia inhibitory factor (LIF) can be utilized. The absence of LIF from the medium or the removal of ESCs from the feeder layer can lead to the development of “embryoid bodies”, which contain all three germ layers: the endoderm, mesoderm, and ectoderm [23,24,25,26,27]. Swift advancements in biotechnology have raised several urgent ethical and policy concerns related to the use of embryonic stem cells in stem cell research, primarily due to the use of human embryos. This has resulted in a search for alternatives that can replace the reliance on human embryonic stem cells in stem cell studies [28,29].

### 2.2. Human Induced Pluripotent Stem Cells

The development of induced pluripotent stem cells (iPSCs) from adult somatic cells was undertaken to address the ethical and immunological concerns related to embryonic stem cells [30]. iPSCs can be characterized as “embryonic stem cell-like” cells produced by reprogramming adult somatic cells by introducing specific pluripotency-related genes. Like embryonic stem cells, iPSCs can extensively proliferate in culture and differentiate into the three primary germ cell layers: endoderm, mesoderm, and ectoderm. This impressive technique of reverting somatic cells to a pluripotent embryonic state while maintaining the same genetic identity is known as somatic cell nuclear transfer (SCNT) [31]. In 2006, Shinya Yamanaka and Kazutoshi Takahashi successfully created mouse iPSCs using a retroviral approach to reprogram mouse fibroblasts by introducing a combination of four transcription factors associated with reprogramming: Oct 3/4 (Octamer-binding transcription factor-3/4), Sox2 (Sex-determining region Y)-box 2, Klf4 (Kruppel Like Factor-4), and c-Myc, collectively referred to as the “OSKM factors” [32]. The following year, in 2007, Yamanaka and his team applied the same reprogramming technique to adult human fibroblasts, resulting in human iPSCs (hiPSCs). Simultaneously, James Thomson’s group also generated hiPSCs using a different delivery method, lentivirus, and an alternative set of four factors: Oct 3/4, Sox2, Nanog, and Lin 28 [33,34]. Significant advancements have been achieved over time to enhance reprogramming efficiency and mitigate the risks associated with this method, such as low reprogramming efficiency, chromosomal instability, and insertional mutagenesis. Innovative strategies employed to enhance reprogramming include the inhibition of obstacles to reprogramming, the use of non-integrative delivery techniques, the overexpression of beneficial genes, and the application of specific molecules that promote reprogramming [35,36,37,38,39,40]. The potential of iPSC technology is substantial for personalized cell-based therapies, human disease modeling, and drug development and testing.

### 2.3. Neural Progenitor or Stem Cells

Neural progenitor cells (NPCs) are multipotent neural stem cells (NSCs) capable of self-renewal and differentiation into neurons and glial cells [41]. However, NPCs do not give rise to non-neuronal cells present in the central nervous system (CNS), such as immune cells. NPCs are found throughout the CNS at various stages of development, including during embryogenesis, in neonatal brains, and in mature adults, indicating that NPCs are not solely embryonic stem cells [42]. NPCs are identified by their specific brain localization, morphological characteristics, gene expression patterns, developmental timing, and functional roles [43]. Additionally, NPCs can be generated in vitro through the differentiation of ESCs or iPSCs using specific neural induction growth factors [44].

## 3. Brain Organoids

**Figure 1:**
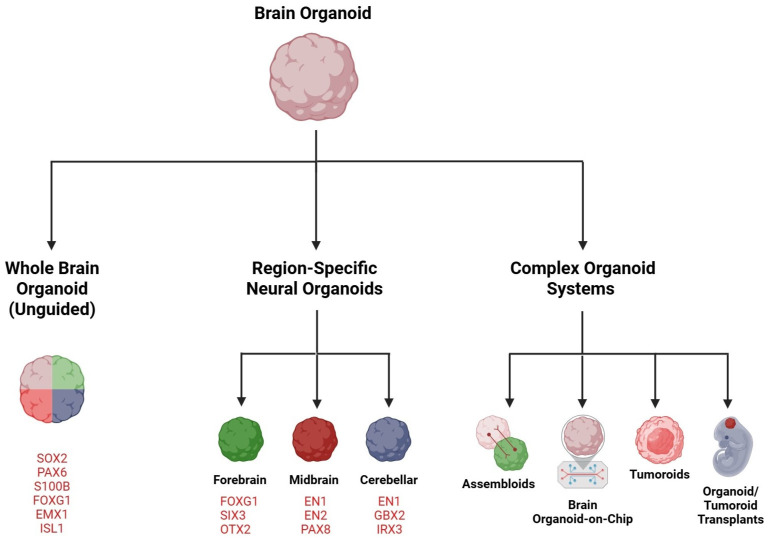
Brain organoids—an overview. Embryonic or induced human pluripotent stem cells (hPSCs) can be differentiated into self-organizing, unguided neural organoids (whole brain organoids) or, by using established differentiation protocols, into neural organoids resembling specific brain regions (guided). Commonly used organoid-specific markers are indicated. Organoids can be combined with other organoids, additional cell types, or hPSCs of various origin into assembloids to construct complex, functional brain structures. Organoids-on-a-chip use bioengineering and microfluidic technologies to enhance cytoarchitecture and long-term organoid culture. Tumoroids are specialized brain organoids mimicking brain tumors with applications in drug discovery, to understand tumorigenesis, or as assembloids, in cancer neuroscience. Organoids and tumoroids are suitable as transplants in mouse brains. Created in https://BioRender.com.

Brain organoids derived from hESCs or hiPSCs can be categorized as cerebral, also known as whole brain organoids or, by consensus, unguided neural organoids [45]. These are usually generated through unguided or minimally guided techniques that depend on self-organizing development and the inherent differentiation abilities of hPSC aggregates. Alternatively, there are region-specific organoids, or regionalized neural organoids, such as those from the forebrain, midbrain, and hindbrain, which are formed using guided methods that involve adding external patterning factors to encourage hPSCs to differentiate into specific lineages [45,46,47]. This section briefly discusses these several types of brain organoids (Figure 1).

### 3.1. Whole Brain or Cerebral Organoid

The groundbreaking achievement of creating cerebral organoids began with discoveries made in 2005 by a research team headed by Yoshiki Sasai [48]. This team was the first to successfully generate 3D neural tissues in vitro that resemble the mouse cerebrum using a cell aggregation suspension culture with mouse embryonic stem cells (mESCs). By 2008, Sasai’s group had refined a differentiation method for cerebral nerve tissues, enabling the 3D differentiation of brain tissues using human ESCs [49]. In the years following the initial reports on neural organoids, the field has witnessed a remarkable increase in their application and ongoing enhancement. Protocols for whole brain organoids depend on the cells’ intrinsic signaling capabilities within the culture, forming various brain regions and CNS structures [4,46,50,51,52]. Although whole brain organoids do not encompass all areas of the brain, they can evolve into multiple neural structures. Since the precise sequence and timing of the necessary signals for specific developmental processes remain unknown, this method allows for observing the patterning of neighboring neural structures, interactions between cells across different brain regions, and characterizing cell morphologies and synapse development. Whole brain organoids, which frequently differ in size and include various cell types with distinct regional identities in a mixed arrangement, have generated interest in utilizing guided differentiation protocols to shape neural structures [53].

### 3.2. Forebrain Organoid

The forebrain represents the largest section of the brain and includes the cerebral cortex, white matter, and corpus callosum. Cortical organoids that replicate the structure of the cerebral cortex have been more thoroughly studied and are used more frequently than other types of brain organoids. These cortical organoids have garnered significant attention due to the unique nature and evolutionary expansion of the cerebral cortex in humans compared to other species, as it is also commonly affected by numerous neurological disorders [54,55,56]. Over the years, various protocols have been developed to generate cortical organoids from stem cells [3,57,58,59,60,61]. Forebrain organoids have been utilized in modeling diseases, such as neurodevelopmental and neurodegenerative disorders and infections like the Zika virus. This article will cover disease modeling using organoids in a later section.

### 3.3. Midbrain Organoid

The midbrain is the uppermost section of the brainstem and includes many significant nuclei and white matter pathways, primarily associated with motor regulation and auditory and visual pathways [62]. Midbrain organoids feature spatially organized clusters of dopaminergic neurons (mDA) that exhibit active synapses, with astrocytes and oligodendrocytes. Several midbrain organoid generation systems have been developed and improved over the past decade [63,64,65,66,67,68,69,70,71]. Organoids that model the human midbrain are particularly noteworthy because this area is explicitly affected by neurodegenerative processes in Parkinson’s disease [72], which will be discussed in detail in the later disease-modeling section.

### 3.4. Cerebellar Organoid

The cerebellum is the largest part of the hindbrain and is home to the highest number of neurons in the human brain. It plays a crucial role in coordinating voluntary movements, motor skills, balance, posture, and speech, as well as contributing to more complex neurological functions like cognition and emotional regulation [73]. This region of the brain may be involved in various human developmental disorders, including autism spectrum disorder, epilepsy, ADHD, and cerebral palsy, and it is recognized as the origin of medulloblastoma, the most prevalent brain tumor in children [74,75,76,77,78,79,80,81,82,83]. Research utilizing brain organoids to investigate cerebellar development has not been extensively pursued. The first comprehensive protocol for creating cerebellar organoids was introduced in 2015 [84], and subsequent studies based on this methodology have been conducted [85,86,87,88,89]. The most recent studies [54,90] succeeded in producing cerebellar organoids that include functional Purkinje cells and enable the prolonged cultivation of all primary cell types found in the cerebellum.

## 4. Complex Organoid Systems

### 4.1. Assembloids

Organoids have enhanced our understanding of organ assembly and how disease can disrupt normal physiology. Nevertheless, they frequently fail to represent the interactions between different tissue types and/or lineages that lead to the emergence of tissue properties during development. Assembloids are fusions of organoids from specific brain regions that aim to replicate the interactions between different regions and cells and the development of neural circuitry, by merging various brain areas and/or cell types. Consequently, assembloids can serve as a tool for modeling nuanced functional disruptions that represent intricate neurodevelopmental, neuropsychiatric, and neurodegenerative disorders [45,91,92,93,94]. Multiple organoids can be combined to create “multi-region assembloids”. These organoids may originate from healthy sources, patient-derived cells, genetically modified hiPSCs, hESCs, or primary tissue sources such as isolated adult stem cells, excised tumors that retain growth potential, or fetal tissue. These assembloids can incorporate two, three, or even more components [93]. Assembloids can be used to study rare diseases, and this will be discussed further in a later chapter.

### 4.2. Brain Organoid-on-a-Chip

Despite the considerable progress made with brain organoids, several limitations remain that impede their acceptance and use by the pharmaceutical industry for modeling neurological disorders and conducting drug tests. One of the key challenges with organoids is the significant variability that can occur, even among those derived from the same stem cells and cultured under identical conditions. Additionally, brain organoids often develop a necrotic core as they mature. This phenomenon arises from an insufficient vascular supply, which hinders the delivery of essential oxygen and nutrients for proper growth [2,95]. In light of these constraints, microfluidic devices are alternative culture systems that have the potential to enhance culture conditions, thereby promoting more consistent organoid development [96,97]. As such, microfluidic cell cultures leverage engineering and technological methods to address biological questions.

Brain organoid-on-chip systems have emerged as a new study area in recent years. These systems combine brain organoid cultivation with microfluidic technologies to enhance culture conditions, increase physiological relevance, ensure reproducibility, and support the industrial applicability of brain organoids. Brain organoids-on-chips are viewed as beneficial for improving culture conditions by minimizing the shear stress that cells endure, enhancing oxygen supply and distribution, facilitating nutrient and waste exchange, and promoting the establishment of chemical gradients [98]. The chips are typically designed in microcolumn, single-channel, or multichannel configurations to provide the physiological, biochemical, and physical signals required to develop brain organoids, thereby promoting their long-term survival and intricate architecture [99].

## 5. Brain Tumoroids

Advancing personalized medicine and addressing treatment resistance in brain tumors have become crucial for effectively translating laboratory research findings into clinical practice [100]. iPSC- or ESC-derived brain organoids themselves may be valuable for studying significant stages of tumor development; however, they do not accurately reflect the unique genetic makeup and cellular and molecular diversity associated with specific tumors and individual patients. Progress in stem cell culture has led to the development of innovative organoid technology, which produces 3D tissues from patient samples in vitro that closely resemble the structures, unique functions, molecular traits, genomic changes, expression patterns, and tumor microenvironments of primary tumors [101]. Tumor organoids, also known as tumoroids, are 3D structures composed of diverse cell types derived from patient samples, replicating the original tumor’s essential histopathological, genetic, and phenotypic features [100]. Brain tumoroids replicate the brain’s structure and function while preserving their histopathological characteristics, genetic makeup, mutation patterns, and responses to treatment [102,103]. Glioblastoma tumoroids are the most used brain tumoroids. Several studies have demonstrated that these models provide several advantages, including the ability to conduct molecular investigations. Additionally, they serve as promising tools for cost-effective studies on new anticancer drugs, which can be applied in precision medicine [104].

## 6. Functional Assays and Omics Used in Brain Organoid Characterization

**Figure 2:**
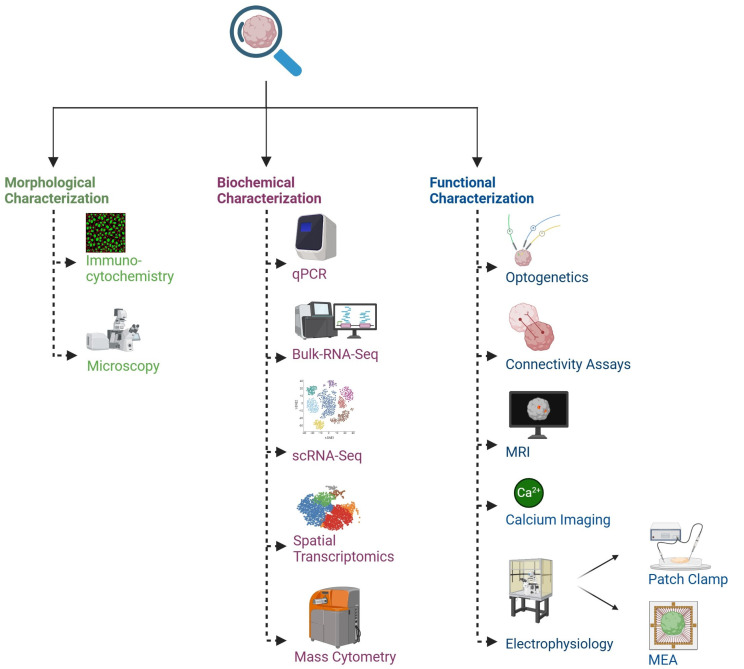
Common methodologies for studying brain organoids and assembloids. Reproducibility, reliability, and translational applicability of neural organoids and assembloids require thorough quality control, standardization, and molecular and functional validation. Created in https://BioRender.com.

Defining the 3D structural arrangement of cells, including the shape, size, and distribution of neurons within organoids, is crucial for identifying similarities with the human brain [105]. The initial characterizations of organoids utilized standard immunohistological markers primarily for identifying cell types, alongside qPCR to evaluate gene expression and determine the composition of cell types; however, comprehensive characterization remained limited [4,51] (Figure 2).

Single-cell RNA sequencing (scRNA-seq) has recently uncovered a notable diversity of cell types present in brain organoids [52]. Understanding this diversity is crucial for enhancing the development and characterization of organoids, particularly in the context of disease modeling [94,106]. Initial organoid characterization lacked depth. Without a clear understanding of neuronal maturity and subtypes, cellular interactions, and functional development, assessing the effectiveness of brain organoids as accurate physiological disease models remained challenging [46,107].

Electrophysiology is the primary method for analyzing the functionality of neural cells and tissues, including brain organoids. The capability to monitor neuronal activity is vital for numerous applications of brain organoids, particularly in disease modeling and drug development [108], and can be achieved using various electrophysiology techniques. The patch-clamping technique enables recording individual neurons’ activity within a brain organoid with high temporal precision, facilitating an in-depth examination of specific neurons [3,108,109]. This high temporal precision is particularly beneficial for assessing how specific disturbances, such as drug treatment or optogenetic stimulation, affect neuronal responses. However, since only single neurons are evaluated, there is a lack of information regarding network connectivity or dynamics that are critical to the overall function of the organoid [106]. On the other hand, calcium imaging addresses the shortcomings of patch clamping by enabling the real-time imaging of neural activity in small neuron clusters. This method is advantageous for investigating particular areas of brain organoids and for analyzing synaptic activity and neural circuits [110]. Research employing confocal or multi-photon microscopy has demonstrated changes in fluorescence resulting from the interaction of Ca^2+^ ions with genetically encoded calcium indicators derived from the aminopolycarboxylic acid BAPTA (1,2-bis(o-aminophenoxy) ethane-N,N,N′,N′-tetraacetic acid) [111]. While calcium imaging enables the functional characterization of individual neurons and small groups, it falls short of comprehensively analyzing dynamic processes occurring at the network level [105].

Microelectrode arrays (MEAs) are becoming more popular for screening applications and various studies because they combine the precise timing of patch clamping with the broader network perspective offered by calcium imaging [112,113,114]. MEAs can simultaneously track responses to stimulation protocols, utilizing a range of electrodes from dozens to thousands [115,116]. Investigating large areas of brain organoids has uncovered significant connectivity between different regions within the organoids, indicating the presence of long-range neural circuits and connectivity between regions. [117]. As brain organoids increase in complexity and are employed to model intricate developmental processes and diseases, the capacity to identify and analyze connectivity and neural circuits between different regions over substantial distances becomes essential, making MEAs valuable tools for understanding these circuits. Recently developed 3D MEAs [118,119] that integrate electrodes into flexible, hinged probes allow for extracellular recordings from 3D neural networks, including those found in organoids. Notably, these devices work with various existing recording systems promoting quick adoption among researchers studying brain organoids.

Optogenetics and the previously mentioned techniques have enabled precise stimulation and mechanistic investigations [120]. Recently, all-optical electrophysiology has become a technique for manipulating and recording neural activity with high spatiotemporal resolution [121,122,123]. The capability to stimulate and record using this approach facilitates network-level recordings of neural circuits. Although it may be slightly less accurate than patch clamping, optical electrophysiology retains much of the resolution offered by patch clamping while enhancing throughput, which is crucial for the extensive analysis required for brain organoids [106]. Recently, a technique utilizing MRI for the deep tissue imaging of whole brain organoids has been developed. This high-resolution imaging has been applied to visualize human brain organoids as small as 2 mm in diameter. MRI and diffusion tractography present a powerful method for imaging organoids. This has the potential to significantly contribute to the investigation of microstructural alterations in brain organoids that model various conditions affecting the human brain and to be used to evaluate neurotoxicity in drug screening [124].

A recent review by pioneers in the field highlighted methods for characterizing brain organoids [94]. This consensus article provides a detailed framework to enhance the reproducibility, reliability, and translational applicability of neural organoid and assembloid models. It specifies recommended practices for generating and characterizing neural cultures derived from hPSCs, such as the thorough quality control of induced iPSC lines, the standardization of differentiation protocols, and suggestions for functional and molecular validation. The authors address the difficulties associated with 3D phenotyping, transplantation into animal models, and ethical considerations, offering essential experimental guidelines for accurately modeling human brain development, disease, and evolution. This work is a key reference for researchers utilizing stem cell-based models in neuroscience, highlighting the importance of transparency, data sharing, and rigorous statistical methods.

## 7. Brain Organoids as Disease Models—Cancer

The emergence of brain organoids derived from human stem cells provides a flexible platform for understanding the molecular and cellular processes involved in brain development, neurodevelopmental disorders, and cancer, thereby connecting fundamental research to therapeutic advancements or personalized medicine [46,125,126,127].

### 7.1. Gliomas

Gliomas are the most prevalent primary brain tumors in adults. Histologically they are similar to normal glial cells and these tumors are commonly named based on these similarities [128,129]. Glioblastoma (GB) is the most common and aggressive type of primary brain tumor in adults. It exhibits distinct histopathological features, including necrosis and endothelial proliferation which contribute to its classification as grade IV according to the World Health Organization (WHO) brain tumor classification [130]. In children, most gliomas are low-grade tumors, typically associated with favorable survival rates [131]. However, approximately 30% of these tumors fall into the high-grade category, which is associated with poorer outcomes and a limited number of long-term survivors [132]. Pediatric high-grade glioma (pHGG) develops in the cerebral hemispheres, known as glioblastoma multiforme (GBM), or in midline structures like the thalamus or brainstem and named diffuse intrinsic pontine glioma (DIPG). Recent molecular studies of pHGG have revealed genetic and epigenetic characteristics that differ from those found in adult gliomas [133]. Existing GBM model systems are inadequate in capturing the complex characteristics of GBM and have not led to sufficiently effective therapies. In the search for new preclinical models for GBM, a variety of 3D organoid-based systems using human cells have been created [134] (Table 1).

Two primary types of brain organoid-based GBM models are derived from clinically relevant stem cells. One of these models enables the examination of GBM initiation and responses to treatments without explicitly accounting for the impact of surrounding tissues or other cell types. This model typically involves the genetic modification of stem cells to incorporate driver genetic changes associated with GBM at a particular point during the differentiation of the cultured organoids. The second model type is often more appropriate for investigating advanced GBM, as it considers its invasion, proliferation, and interactions with other cell populations in the brain. These models can be created from a blend of glioblastoma stem-like cells and organoids derived from PSCs. Alternatively, they may consist of co-cultures of GBM cells, which can be either patient-derived primary cells or established GBM cell lines, prepared in single-cell suspensions or as spheroids [167,168]. A recent study produced engineered GBM brain organoids using genetically altered stem cells with mutations from both the proneural and mesenchymal subtypes of GBM. The research revealed that mutations linked to GBM disrupt neural development within brain organoids. Furthermore, when cells from engineered GBM brain organoids were transplanted into mice, they formed tumors that mimicked the specific characteristics of human GBM tumors. By utilizing integrated single-cell transcriptomic data from engineered GBM brain organoids and patient-derived GBM tumors, this research team uncovered shifting gene expression patterns in developmental cell states contributing to tumor progression [135]. A different study effectively generated patient-derived GBM organoids that maintained the pathological characteristics of the parental GBM. The patient-derived GBM organoid–neuron co-culture system was also explored, and it revealed interactions between neurons labeled with GFP and patient-derived GBM organoids labeled with mCherry. The in situ stereotaxic implantation of patient-derived GBM organoids into the brains of nude mice established intracranial orthotopic GBM models [136].

Deligne and colleagues developed an integrated method to produce, expand, and thoroughly analyze 3D pediatric high-grade glioma-derived organoids from fresh tissues for precision and personalized medicine assays. They generated 3D patient-derived tumor organoids from diverse primary and relapsed histological subtypes and tumor grades of pHGG. The generated 3D models were biobanked and revived for additional molecular, cellular, and preclinical studies, achieving a success rate of 91% [169]. A different study revealed the successful creation of a neuroimmune-competent brain organoid model composed entirely of human cells that effectively represents multiple critical diffuse midline glioma (DMG) features. They generated human microglia-containing brain organoids derived from genetically modified hPSCs. By fusing these microglia-containing organoids with DMG spheroids, they established a manageable and modular platform for investigating interactions between human tumors and the brain within a 3D microenvironment. The microglia brain organoids contained microglia exhibiting a mature morphology and motility, alongside various neuron subtypes, simulating the intricate microenvironment in brain tumors. Subsequently, these organoids were combined with tumor spheroids harboring H3K27M mutations and TP53P27R/K132R alterations to form a microglia-containing brain organoid-tumor fusion model. The results of this study suggested that DMG cells interacting with another DMG spheroid show restricted infiltrative activity, preserving a distinct cellular boundary over a prolonged period [137]. A recent review extensively discussed organoid models of glioblastoma [167].

### 7.2. Medulloblastoma

Medulloblastoma is a highly aggressive brain tumor that originates from the cerebellum, primarily affects children, and is responsible for a significant proportion of morbidity and mortality among cancer patients [170,171]. Medulloblastoma exhibits biological and clinical diversity, comprising several subgroups [171,172]. Over the years, continuous efforts have been made to develop models of this disease that accurately replicate in vivo tumors. This has led to the growing use of cerebellar organoids for modeling medulloblastoma. A recent investigation utilizing the CRISPR/Cas9 gene editing of Patched-1 (PTCH1) in hiPSCs led to the generation of a medulloblastoma-cerebellar organoid [138]. PTCH1 serves as the primary receptor for the Sonic Hedgehog (SHH) ligand and plays a crucial role in inhibiting SHH signaling, which is vital for human embryonic development. Mutations that result in its loss of function have been linked to disrupted neuronal growth and the aggressive brain tumor, medulloblastoma. The resulting organoids were employed to explore the initial molecular and cellular effects of PTCH1 mutations on the development of the human cerebellum. The results from these studies indicated that the developmental processes occurring in cerebellar organoids mirror the in vivo mechanisms of regionalization and SHH signaling, providing new perspectives on the early pathophysiological stages involved in the tumorigenesis of medulloblastoma without relying on animal models [138,139].

One study aimed at understanding the phenotypic traits of SHH medulloblastoma in a nonmalignant tumor microenvironment using SHH medulloblastoma cells co-cultured with cerebellar organoids to promote a malignant phenotype. Tumor spheroids from two different SHH medulloblastoma cell lines were grown alongside control organoids. The investigators analyzed the transcriptional profiles of the co-cultured tumor cells and compared them to those from mono-cultures, patient tumors, and patient-derived orthotopic xenograft (PDX) mouse models. The co-cultured SHH medulloblastoma cells displayed phenotypes akin to actual patient tumors and showed greater heterogeneity compared to mono-cultures. Some cell subpopulations activated NEUROD1, a marker for differentiating granule cells, which was not present in mono-cultures. Other subpopulations demonstrated traits of a cancer stem-like state like those found in vivo. This study introduced a novel in vitro model of SHH medulloblastoma with promising translational potential [139].

Another study used cerebellar organoids to model the Group 3 medulloblastoma subtype, which among medulloblastoma has the poorest prognosis. The investigators overexpressed c-MYC and Otx2, which are recognized as significant promoters of this subtype. Initially, they pre-differentiated the cerebellar organoids until day 35, ensuring all progenitors were present, and subsequently transfected the organoids with the oncogenes. This research employed a transposon system to enable the ongoing overexpression of the targeted genes over time, replicating the oncogene amplification and overexpression seen in patients. The resulting medulloblastoma-like organoids exhibited a DNA methylation profile that aligns with human Group 3 tumors. Furthermore, this study demonstrated that SMARCA4 can mitigate the tumorigenic effects of Otx2 and c-MYC in vivo and that treatment with Tazemetostat, an EZH2-specific inhibitor, diminishes Otx2/c-MYC tumorigenesis both in ex vivo cultures and human cerebellar organoids [140].

## 8. Brain Organoids as Disease Models—Neurodevelopmental Disorders

### 8.1. Autism Spectrum Disorder

Autism spectrum disorder, known as ASD, is a complex neurodevelopmental condition that arises in developing fetuses and young children during their early growth stages. It is a multifactorial condition that in part is attributed to changes in the expression of specific genes [173,174]. ASD affects more than 2% of the preschool population and encompasses a significant range of conditions that contribute to the heterogeneity of autism [175]. The absence of a suitable human disease model hampers research efforts to effectively replicate human pathological features and reflect the genetic diversity associated with ASD [147,176,177,178,179]. Extensive studies have been conducted using brain organoids to model and investigate the molecular basis of autism (Table 1). One of the most recent studies utilized iPSCs and cerebral organoids derived from three individuals with idiopathic non-regressive ASD who presented with normocephaly [141]. This study demonstrated that neural stem cells in the ASD organoids exhibited premature differentiation and impaired expression of fatty acid binding protein 7 (FABP7). Furthermore, it was determined that FABP7 operated through the Mitogen-Activated Protein Kinase Kinase1/2 (MEK1/2) pathway to facilitate early neurogenesis. This discovery offers new insights into the molecular mechanisms involved in the pathology of idiopathic normocephalic ASD with non-regression and encourages the development of therapeutic drugs aimed at ASD treatment by targeting the FABP7/MEK axis [141].

A different study established a human cerebral organoid model using induced pluripotent stem cells, applying targeted genome editing to eliminate the protein expression of the CNTNAP2 gene. Homozygous mutations leading to the loss of function in CNTNAP2 are linked to cortical dysplasia focal epilepsy syndrome, which includes features of autism spectrum disorder (ASD) and intellectual disability [180]. In CNTNAP2−/− cerebral organoids, an accelerated cell cycle, disorganized ventricular zones, and increased cortical folding were shown. Proteomic studies revealed disruptions in glutamatergic and GABAergic synaptic pathways, while transcriptomic analysis showed altered gene expression related to inhibitory neuron networks. Additionally, there was heightened AKT/mTOR signaling observed in these organoids. A spatial transcriptomic analysis of the CNTNAP2−/− ventricular-like zones revealed gene expression alterations, indicating the upregulation of pathways related to cell cycle regulation, synapses, and glutamatergic/GABAergic activity. A significant overlap was discovered among the differentially expressed genes from day-30 organoid omics datasets and those from idiopathic ASD (macrocephaly) iPSC-derived telencephalic organoids [142].

Tanabe’s group utilized patients-derived organoids to investigate whether the onset of ASD is linked to an underdeveloped choroid plexus [143]. The choroid plexus consists of multiciliated epithelial cells that differentiate from ependymal cells lining the ventricular surfaces [181]. It generates protein-rich cerebrospinal fluid and completes its maturation before brain development. It is believed that dysfunction in the choroid plexus significantly impacts ASD. Over time, an increase in the expression of transthyretin (TTR), a marker gene for the choroid plexus, was detected in choroid plexus organoids sourced from healthy individuals. Additionally, epithelial cells that expressed TTR exhibited an aggregated signal at the apical surface, resembling the intrinsic multicilia found in choroid plexus epithelial cells. Conversely, choroid plexus organoids derived from ASD patients displayed a decrease in TTR expression for one patient. The expression levels of the neural progenitor cell marker Pax6 and the neuronal marker β3-tubulin showed no significant difference, suggesting that the developmental abnormalities in the choroid plexus emerged later in the differentiation process. When evaluating organoid size at the choroid plexus differentiation stage (day 50), the surface area of organoids from ASD patients was significantly smaller, half the size. The assessment of ciliogenesis and protein expression (TTR, Otx2, AQP1, and ZO-1) in organoids from ASD patients showed a marked reduction in these genes. These findings suggested that a choroid plexus exhibiting impaired multiciliogenesis and a reduced expression of key differentiation genes affects blood cerebrospinal fluid barrier integrity and contributes to dysfunction in organoids from individuals with ASD. Overall, this study highlighted that an underdeveloped choroid plexus is a prevalent characteristic in ASD pathology [143].

SHANK (SH3 and multiple ankyrin repeat domains) proteins act as scaffolds at synapses and are primarily found in the post-synaptic density of excitatory synapses. Changes in SHANK3 have been closely linked to non-syndromic ASD. Research using cerebral organoids to observe the developmental changes in SHANK3 expression identified the binding site of the upstream Early Growth Response 1 (EGR1) in the promoter region of the SHANK3 gene. The validation of this interaction through chromatin immunoprecipitation (ChIP) and electrophoretic mobility shift assays (EMSAs) confirmed that EGR1 interacts with the promoter region of SHANK3, and alterations in EGR1 levels affect SHANK3 transcription. This highlights the important link between EGR1 and SHANK3 in the context of ASD [144].

SCN2A encodes the Nav1.2 voltage-gated sodium channel and is significantly reduced in individuals with autism [182]. Wu et al. [145] explored the role of microglial phagocytosis in synapses and found that microglia are integral to the pathogenesis of autism in SCN2A-deficient mouse and human cerebral organoids models involving excessive phagocytic activity. The capacity of microglia to eliminate surplus synapses from affected neurons is evolutionarily preserved and has been seen in rodent and human cellular models with SCN2A deficiency [145].

A separate investigation utilized forebrain organoids to delve into germline research. Mutations in the PTEN gene account for 0.2–1% of all cases of ASD and around 17% of ASD patients with microcephaly. This study used forebrain organoids from gene-edited isogenic human iPSCs containing PTEN mutant alleles linked to ASD or cancer to investigate how these mutations interfere with neurodevelopmental processes. The results indicated that the ASD allele hindered early neuroectoderm formation, leading to impaired electrophysiological function. Organoids derived from cancer-related iPSCs exhibited disturbances in neuronal differentiation, radial glia positioning, and cortical layering after 72 days of development. Perifosine, an AKT inhibitor, diminished the overly activated AKT and partially rectified the cellular organization abnormalities observed in the ASD organoids. ScRNA-seq analyses of early-stage organoids indicated a gene reduction associated with neural cell fate in the ASD mutant organoids [146].

Genes associated with chromatin remodeling frequently show mutations in individuals with ASD. One of the most affected and penetrant genes is chromodomain helicase DNA-binding protein 8 (CHD8). It is characterized by a SFN2-like ATPase and contains two chromo-domains. Most ASD-associated de novo mutations in CHD8 result in the loss of function, leading to gene haploinsufficiency. CHD8 haploinsufficiency is associated with the abnormal development of the cerebral cortex. Using cerebral organoids, it was found that CHD8 haploinsufficiency disrupted neurodevelopmental pathways, causing the accelerated generation of inhibitory neurons and the delayed generation of excitatory neurons, resulting in opposing proportional expansions at days 60 and 120 [147]. This imbalance correlated with an increase in the size of the cerebral organoids, serving as an in vitro representation of macrocephaly observed in patients [183]. Another group investigated changes in forebrain development by utilizing cortical organoids from boys with idiopathic ASD and their unaffected fathers across a study involving 13 families. Thus, the development of ASD in both macrocephalic and normocephalic individuals involves the imbalance of excitatory neurons in the dorsal cortical plate and other cell types, stemming from divergent transcription factor expression during early cortical development. Although no genetic variants were found in the probands linked to the gene expression changes, a significant overlap with known ASD risk genes suggested a genetic convergence between rare ASD types and idiopathic cases [148].

A study by Birtele et al. [149] utilized cortical organoids to investigate the non-synaptic roles of the ASD-related gene SYNGAP1 (synaptic Ras GTPase-activating protein 1). Evidence suggests that SYNGAP1 may have a significant function during the early phases of cortical neurogenesis. The study revealed abnormal cytoskeletal dynamics that disrupt the scaffolding and division orientation of human radial glial cells, leading to impaired lamination and the hastened maturation of cortical projection neurons in cortical organoids with SYNGAP1 haploinsufficiency [149].

ASD is complex and multifaceted. Currently, over 800 genes and dozens of genetic syndromes have been associated with ASD, with environmental and epigenetic factors further adding to this complexity [184]. The work outlined above and additional studies [179,185,186,187,188,189,190,191,192,193,194,195,196] extensively summarized in [183] demonstrate the immense value brain organoids bring to modeling autism, thereby greatly enhancing our understanding of the molecular underpinnings of this neurodevelopmental condition.

### 8.2. Epilepsy

Epilepsy is a condition that includes various underlying disorders [197]. The defining characteristic of epilepsy is seizures [198]; it manifests in different types. In focal epilepsy, seizures originate from a specific region of the brain, while in generalized epilepsy, seizures arise simultaneously on both sides of the brain. Epilepsies are classified according to their causes, which can be either acquired or genetic [199]. Roughly 30–40% of individuals with epilepsy do not achieve the total control of their symptoms through antiepileptic medication [200]. The introduction of next-generation sequencing has led to the discovery of over 140 genetic loci linked to various forms of epilepsy. Genes linked to epilepsy encode various proteins, ranging from ion channels to transcription factors. While the specific pathways through which mutations lead to seizures are not fully understood, they ultimately enhance brain excitability [201]. Conventional methods for modeling genetic forms of epilepsy have relied on rodent models created through genetic alteration or by selecting spontaneous mutations in rodent populations. These models often fail to represent the human condition and its underlying mechanisms [202,203]. The use of brain organoids for studying epilepsy and similar disorders is becoming more prevalent, underscoring their increasing relevance in epilepsy research.

Focal cortical dysplasia (FCD) stems from abnormal neural progenitor cell proliferation and tissue organization during brain development, and is linked with developmental delays, reduced cognitive function, and persistent epilepsy [204,205]. The primary pathogenic factor for FCD II is thought to be somatic mosaicism affecting genes involved in the mTOR pathway, occurring in cells such as cortical neurons in the brain, which leads to localized abnormal cortex growth [206]. Loss of function in specific genes, which includes GATOR1 complex subunits, including DEPDC5, NPRL2, and NPRL3, leads to the activation of the mTOR pathway [207,208]. Cortical organoids from FCD II patients were larger than those from the control. This finding aligns with the hyperactivation of the mTOR signaling pathway and abnormal growth observed in patients with FCD [150].

Another recent study focused on PCDH19-clustering epilepsy (PCE). This arises from pathogenic variants in the Protocadherin-19 (PCDH19) gene, located on the X chromosome [209]. This condition primarily affects females and mosaic males. The mosaic expression of the cell adhesion molecule PCDH19 is believed to disrupt interactions between cells expressing mutant and wild-type PCDH19, leading to the development of the disorder. In this study, the investigators used genome editing to create isogenic female human ESCs featuring HA-FLAG-tagged PCDH19 (wild-type) or homozygous PCDH19 knockout. They generated human cortical organoids from mixed GFP-labeled wild-type and RFP-labeled knockout cells. The study found that PCDH19 was present in early wild-type neural rosettes (days 20–35) and co-localized with N-Cadherin in areas like the ventricular zone. In mosaic PCE, abnormal cell sorting in the ventricular zone was noted, which continued even with different ratios of wild-type to knockout cells. PCE cortical organoids exhibited variations in PCDH19 and N-Cadherin expression and irregular deep-layer neurogenesis. These issues were absent in organoids made only from wild-type or knockout cells, simulating male carrier scenarios. The outcomes from these mosaic PCE human cortical organoid models suggest that PCDH19 is crucial for the organization of radial glial cells in the human ventricular zone and for early cortical development. Thus, this model may serve as an essential platform for investigating the mechanisms behind PCE-related cortical hyperexcitability and evaluating potential precision therapies [151].

Glucose Transporter 1-Deficiency Syndrome (GLUT1-DS) is a rare genetic condition caused by mutations in the GLUT1 gene leading to reduced brain metabolism and symptoms including epilepsy, motor impairments, and cognitive challenges [210,211]. Investigators generated brain organoids from iPSCs derived from a GLUT1-DS patient and healthy control. The functional assay results indicated a comparable distribution of neurons and astrocytes in both organoids. However, the GLUT1-DS organoids displayed lower cellular density and smaller sizes. Both organoids also showed characteristic functional spikes, but the GLUT1-DS organoids exhibited unique epileptiform activity and increased sensitivity to glucose deprivation. Overall, the results from this study supported the application of brain organoids as a model for investigating GLUT1-DS and emphasized their potential for evaluating new therapeutic approaches to enhance glucose metabolism and address epilepsy in patients [152].

Tuberous sclerosis (TSC) is categorized among cortical development malformations arising from mutations in either TSC1 (hamartin) or TSC2 (tuberin) that are components of the mTOR pathway [212]. Patients experience debilitating neuropsychiatric symptoms that are frequently resistant to treatment, including uncontrollable epileptic seizures and intellectual disability. The key features of TSC include cortical tubers—abnormal neurons and glial cells in the cortex associated with seizures. More than three out of four individuals with TSC develop subependymal nodules (SEN). Research indicates that cortical tubers and SEN/SEGAs (subependymal giant cell astrocytoma) typically arise from similar genetic alterations, often characterized by the loss of the second allele of either TSC1 or TSC2 somatic mutations leading to TSC onset that have been observed in both mouse and human models, highlighting the critical role of mutations in NPCs. This randomness may explain the variability in tuber number and size in TSC patients. A higher tuber load correlates with more severe epilepsy and intellectual disability, indicating that somatic mosaicism might significantly impact the differences in neurological symptoms among TSC individuals [213]. Genetic analyses reveal that SEN/SEGAs typically lose the second allele, whereas this is less common in cortical tubers, which raises questions about the two-hit hypothesis. These differences could stem from specific human brain cell types and processes crucial for disease development. In one study, Eichmuller et al. [153] created human cerebral organoids from patients’ iPSCs and compared these to primary human tissues. The study identified a type of neural stem cells called CLIP cells, which proliferate excessively in TSC cases, resulting in excessive interneurons, brain tumors, and cortical malformations. The investigators further explored various therapeutic strategies for TSC and related disorders, including blocking the EGF receptor, which has shown promise in reducing tumor burden. This work also showed that examining cortical malformation can yield valuable insights into unique aspects of human brain development [153]. Other studies [214,215,216] that utilized brain organoids to study disorders characterized by epilepsy explore the fundamental mechanisms and possible treatment strategies related to these conditions.

### 8.3. Cerebral Palsy

Cerebral palsy (CP) affects movement and posture, reducing activity and functionality due to non-progressive damage to the developing brain [217]. It is the most prevalent physical disability affecting children in the United States [218,219]. Over the years efforts have been made to develop more realistic models for studying CP, including brain organoids. However, using brain organoids in CP research presents significant challenges, particularly because the specific cause of CP remains unknown in about 80% of patients.

TYW1, an enzyme responsible for tRNAphe modifications, regulates the proliferation and migration of neurons during early neural development [220]. A deficiency in TYW1 could disrupt the accurate pairing of tRNAphe with its respective codons, resulting in reduced levels of proteins rich in phenylalanine. The accurate translation of proteins is essential for neural development [221]. The dysfunction of TYW1 has been associated with CP associated with significant intellectual disabilities. Using cerebral organoids derived from TYW1−/− hESCs, studies showed that the depletion of TYW1 led to the impaired differentiation of neurons. The loss of TYWI led to the increased expression of human-specific endogenous retrovirus-K (HERVK) in this model due to the decreased levels of SMARCAD1, thereby hindering neuron differentiation [154].

Another study focused on neonatal hypoxic injury (NHI), a severe condition impacting over 60% of infants born with a very low birth weight. The condition leads to long-term neurological issues such as seizures, cerebral palsy, and intellectual disabilities [222,223]. The investigators demonstrated the effectiveness of minocycline for treating human NHI using a cerebral organoid model of hypoxia where they examined the development of an in vitro cerebral organoid model under varying oxygen levels. The findings indicated that hypoxia reduced the expression of critical gene markers associated with the development of the forebrain, oligodendrocytes, glial cells, and cortical layers, as well as genes essential for migrating cortical neurons. Conversely, ventral markers showed no change and even increased under hypoxic conditions. The detrimental impact of hypoxia on dorsal brain genes, and on oligodendrocytes and neuronal progenitors, could be alleviated by administering minocycline, a small molecule approved by the FDA [155].

One study developed a GPAM knockout line; GPAM (glycerol-3-phosphate acyltransferase 1, mitochondrial) is an enzyme in the mitochondria that synthesizes glycerolphospholipids and triacylglycerols. Mutations that result in the loss of GPAM function have been linked to hypomyelination in the corticospinal tract of patients with cerebral palsy [220]. The investigators created a GPAM knockout human ESC line, SYSUe-008-A, using CRISPR/Cas9 technology to investigate this rare disorder in human brain organoids. The GPAM knockout cell line retained a normal karyotype and exhibited pluripotent stem cell markers and differentiation capabilities similar to wild-type hESCs, making it a valuable resource for modeling the disease in brain organoids and conducting drug screening for cerebral palsy [224].

### 8.4. Attention Deficit Hyperactivity Disorders

Attention-deficit/hyperactivity disorder (ADHD) is a neurodevelopmental condition marked by inattention, impulsivity, and hyperactivity. It is often identified in early childhood and persists into adulthood [225,226]. ADHD ranks among the most genetically inheritable neuropsychiatric conditions [227,228]. One study utilized brain organoids to investigate the origins of ADHD within the early cerebral cortex, particularly the telencephalon. The investigators created an ADHD organoid using patient-derived iPSCs from an 18-year-old male with ADHD according to DSM-IV-TR criteria. It was found that telencephalon organoids exhibited less growth in their layer structures than control organoids with a higher neuron count but reduced cell proliferation between days 35 and 56, resulting in a notable difference. These alterations in neural stem cell characteristics and cortical structure development may be significant for understanding ADHD’s pathogenesis and align with findings from neuroimaging studies [156].

## 9. Assembloids and Rare Diseases of the Nervous System

Dysfunctions in the neural circuits of the cortico-striatal pathway are implicated in neurodevelopmental disorders such as ASD, schizophrenia, and obsessive–compulsive disorder [229,230,231,232]. To test this, investigators created brain organoids that mimic the developing human striatum and combined them with cerebral cortical organoids in a 3D culture to form cortico-striatal assembloids. The results showed cortical neurons extending axonal projections into striatal organoids and establishing synaptic connections. Following the assembly, medium spiny neurons exhibited maturation in their electrophysiological properties and showed calcium activity when cortical neurons were stimulated optogenetically. The investigators also generated cortico-striatal assembloids from individuals with a neurodevelopmental disorder linked to a deletion on chromosome 22q13.3. This work highlighted disease-associated abnormalities in calcium activity, suggesting that this approach facilitates the exploration of cortico-striatal connectivity development using patient-derived assembloids [157].

Similarly, a recent study generated a striato-nigral (Str-SN) circuit, consisting of medium spiny neuronal projections from the striatum to the midbrain’s substantia nigra (SN). Impairments in the Str-SN circuitry can lead to various motor disabilities linked to neurodegenerative conditions, such as Huntington’s disease. The investigators created 3D Str-SN assembloids by combining striatum-like organoids with midbrain SN-like organoids. Significant medium spiny neuron (MSN) projections from the striatum formed synaptic connections with GABAergic neurons in SN organoids and demonstrated optically evoked inhibitory post-synaptic currents. This study’s results indicated that treatment with brain-derived neurotrophic factors could improve the defects in reciprocal projections within HD-iPSC-derived assembloids. Thus, Str-SN assembloids may be utilized to identify defects in MSN projections and serve as potential platforms for drug testing in Huntington’s disease [158].

Neuronal connections facilitate muscle activation and movement generation. Andersen et al. [159] created 3D cortico-motor assembloids by combining organoids that replicate the cerebral cortex or hindbrain/spinal cord with skeletal muscle spheroids. Techniques such as rabies tracing, calcium imaging, and patch-clamp recordings revealed that corticofugal neurons established links to spinal spheroids, while spinal motor neurons connected with muscle tissue. The uncaging of glutamate or optogenetic activation of cortical spheroids elicited significant contractions in 3D muscle, and these assembloids remained morphologically and functionally intact for as long as 10 weeks post-fusion. This approach highlights the remarkable self-assembly of 3D cultures to form functional circuits for studying development and diseases [159].

Assembloids have been combined with CRISPR screening to explore the roles of the significant number of genes related to neurodevelopmental disorders in the development of human interneurons. The first screen aimed at interneuron generation revealed thirteen candidate genes. A follow-up screen for interneuron migration in over 1000 forebrain assembloids pinpointed 33 potential candidates comprising cytoskeleton-associated genes and the endoplasmic reticulum gene LNPK. The results demonstrated that LNPK deletion led to the irregular migration of interneurons. These findings further emphasized the effectiveness of the CRISPR-assembloid platform in systematically linking neurodevelopmental disorder genes to human development and uncovering the mechanisms behind diseases [160].

Two other studies highlighting the importance of interneurons were conducted by Birey at al. [233,234] in a model of Timothy syndrome, a neurodevelopmental condition associated with mutations in the CaV1.2 calcium channel. Nervous system development involves a coordinated series of events, particularly the movement of GABAergic neurons from the ventral to the dorsal forebrain and their integration into cortical circuits. The investigators created forebrain organoids that included cortical glutamatergic and GABAergic neurons to replicate the migration patterns of interneurons seen during fetal development. They found that interneurons displayed unusual migratory behavior in Timothy syndrome and demonstrated that, post-migration, these interneurons successfully integrated with glutamatergic neurons, forming a functional microphysiological system [233]. Notably, the abnormality in saltation length was associated with irregular actomyosin and myosin light chain (MLC) phosphorylation, while the alteration in saltation frequency was influenced by heightened sensitivity to GABA, which could be corrected by GABA-A receptor antagonists. The study further demonstrated an increase in hypersynchronous hCS network activity in Timothy syndrome that was intensified by interneuron migration. Collectively, these studies highlighted the intricate role of L-type calcium channel Cav1.2 function in the migration of human cortical interneurons and identified new approaches to remediating deficiencies in the context of Timothy syndrome [234].

Many organoid protocols still overlook microglia, which are essential for brain development and have been connected to various neuroinflammatory conditions. One study introduced an innovative method to create neuroimmune assembloids using iPSC-derived cortical organoids and microglia. The authors proposed two approaches: one that directly incorporated microglia progenitors into the organoids and another that combined microglia with neuronal progenitors in a specific ratio [161]. Another study highlighting the need to incorporate immune cells into assembloids was conducted by Goldberg et al. [235]. Mutations in the Three Prime Repair Exonuclease 1 (TREX1) gene have been linked to Aicardi–Goutières Syndrome (AGS). In this study, the investigators employed three-month-old assembloids that included microglia and oligodendrocyte-containing organoids to examine the roles of TREX1 in human microglia. Functionally, TREX1 was essential in microglia during the shift from gliogenic intermediate progenitors referred to as pre-oligodendrocyte precursor cells to precursors in the oligodendrocyte lineage, which hampers oligodendrogenesis while favoring astrogliogenesis in human assembloids. The findings further revealed that TREX1 affects cholesterol metabolism, resulting in a more active microglial morphology and heightened phagocytosis when TREX1 is absent. Regulating the cholesterol metabolism using atorvastatin, an FDA-approved HMG-CoA reductase inhibitor, restored microglial characteristics, suggesting potential therapeutic strategies for diseases like AGS [235].

Studies on Parkinson’s disease (PD) highlight some of the challenges associated with using organoids to study neurodegenerative diseases. Traditional brain organoids from iPSCs require lengthy maturation periods and may not accurately represent the older brain’s epigenetics, limiting their use in modeling late-onset diseases like PD. Kim et al. [236] presented a method for creating 3D-induced dopaminergic (iDA) neuron organoids directly from human fibroblasts, which better mimic the aging brain. The 3D iDA neuron–astrocyte assembloids demonstrated the importance of glial cells in neurodegenerative processes. In PD assembloids, control astrocytes alongside A53T mutant iDAs protected neurons, while A53T mutant astrocytes led to neuronal degeneration and synucleinopathy. The findings suggested that 3D-iDA organoids and assembloids are valuable models for studying PD’s pathogenesis and potential therapies [236]. Another study that generated an assembloid model of PD presented a model of the nigro-striatal pathway using midbrain–striatum assembloids with inducible aging. The findings demonstrated that these assembloids can establish nigro-striatal connectivity, characterized by catecholamine release from the midbrain to the striatum and the formation of synapses between the midbrain and striatal neurons. In addition, assembloids that overexpress progerin exhibited aging characteristics that lead to early signs of neurodegeneration. Thus this model may aid in elucidating the roles of aging and nigro-striatal connectivity in the onset and progression of PD [162].

Synaptic plasticity mechanisms, such as long-term potentiation (LTP) and long-term depression (LTD), are essential for learning and memory. A study by Patton et al. [163] created thalamocortical assembloids by merging hiPSC-derived organoids from the thalamus and cortex. Single-nucleus RNA sequencing revealed that over 80% of thalamic organoid cells were glutamatergic neurons. These assembloids formed reciprocal axonal projections and synapses, displaying short-term plasticity like animal models. While both long-term potentiation (LTP) and long-term depression (LTD) were induced, the underlying mechanisms varied from those observed in rodents. Therefore, thalamocortical assembloids serve as a useful model for investigating synaptic plasticity within human neural circuits [163]. Dysfunctions in thalamocortical communication may play a role in neuropsychiatric conditions. Variants in CACNA1G, which encodes a T-type calcium channel in the thalamus, are associated with absence seizures, intellectual disabilities, and schizophrenia. Research revealed that the M1531V CACNA1G variant linked to seizures caused changes in T-type currents in thalamic neurons and increased activity in both thalamic and cortical neurons. Conversely, the loss of CACNA1G, associated with a higher risk of schizophrenia, resulted in abnormal connectivity in the thalamocortical pathway and heightened spontaneous activity in the thalamus. Overall, these studies highlighted the potential of multicellular systems to study genetic risk variants for diseases at the cellular and circuit levels [46,164].

Assembloids representing different organ systems underscore the future potential of complex assembloid systems. For example, cortical–blood vessel assembloids created by merging cortical organoids with blood vessel organoids enable vascular support for brain organoids. This led to a notable increase in the expression of microglia and astrocytes within the brain organoids in a study aimed at finding the correlation between COVID-19 and Alzheimer’s disease (AD) [165]. With a focus on the neurotrophic effects of SARS-CoV-2, the research identified AD pathologies, such as β-amyloid plaques, which were influenced by the inflammatory responses triggered by SARS-CoV-2 infection. Overall, the assembloid system offered a sophisticated platform for examining human neurotrophic diseases, such as COVID-19, and implied that the neuroinflammation resulting from viral infections plays a role in the advancement of AD [165].

New research into the capacity of the Zika virus (ZIKV) to infect and eliminate CNS tumors such as GBM, medulloblastoma, and Atypical Teratoid Rhabdoid Tumors (ATRTs) highlights the virus’ potential for oncolytic therapy. There is limited evidence demonstrating the safety of ZIKV for treating malignant CNS tumors, which is necessary for advancing this strategy to clinical trials especially in children. Ferreira et al. [237] created a co-culture model using mature human cerebral organoids combined with tumor cells from GBM, medulloblastoma, or ATRT. These models provided the means to monitor the growth and invasion of tumor cells within cerebral organoids and utilized the assembloids to assess the oncolytic effects of ZIKV, its replication abilities, and its preferential targeting of cancer cells over normal cells. The results suggested that ZIKV replicates more rapidly in aggressive CNS tumor cells than in normal cells found in cerebral organoids. However, the elevated replication of ZIKV in tumor cells did not directly correlate with oncolytic effects, indicating the involvement of internal and external cellular factors in tumor cell death by ZIKV [237]. Overall, these studies not only highlight how assembloids aid in investigating rare nervous system diseases but also emphasize the immense potential of merging brain organoid methodology with brain cancer research in the newly emerging field of cancer neuroscience.

## 10. Brain Organoids in Drug Discovery

The application of brain organoids has advanced basic research, especially in modeling diseases and identifying targets related to neurodevelopmental, neuropsychiatric, and neurological conditions [238]. Despite the promise of organoid technology in drug screening, several challenges have emerged concerning its reproducibility, scalability, and relevance to human diseases. To improve the utility of organoids in drug discovery, several technical strategies can be considered, such as using CRISPR to create isogenic models, applying single-cell RNA sequencing for detailed cellular profiling, and leveraging machine learning to process intricate datasets. Additionally, tools like high-content imaging, automated liquid handling, and standardized assays are crucial in facilitating this goal [239]. Robots and software designed to identify organoids in microwells and conduct quantitative imaging analysis facilitated the phenotyping of organoids. This lays a promising foundation for high-quality HTS and potential use as a tool for therapeutic screening [238]. For example, Renner et al. implemented a fully automated HTS workflow for the creation and assessment of midbrain organoids. The researchers utilized a liquid-handling robot capable of executing processes such as seeding, maintenance, fixation, whole-mount staining, and clearing in a fully scalable automated manner within standard 96-well plates. The midbrain organoids generated showed minimal differences in size distribution, morphology, and cellular composition within and between batches, displaying comparable morphological characteristics to those reported in other studies. Further analysis through RNA sequencing and quantitative whole-mount high-content imaging (HCI) validated the reproducibility of these organoids [69]. Another investigation presented a compelling, network-based platform for drug screening, developed by merging mathematical modeling and the pathological characteristics of Alzheimer’s disease with human iPSC-derived cerebral organoids, including isogenic lines modified using CRISPR-Cas9. They utilized 1300 organoids sourced from 11 individuals to establish a high-content screening (HCS) system and evaluate FDA-approved drugs that can penetrate the blood–brain barrier. This research offers a pathway for precision medicine by integrating mathematical modeling with a small-scale pathological brain model using cortical organoids [240]. However, while advances in integrating organoids into drug discovery setups have been made (Table 2), significant challenges in standardization, scalability, and cost remain even beyond those of standard 3D cell culture [241,242].

## 11. Discussion

Brain organoids have become valuable tools for modeling neurodevelopmental disorders, brain tumors, and the development of new therapeutics. However, several issues must be addressed to unlock their full potential for clinical applications. One notable challenge with current organoid models is that they often lack genetic diversity [248]. Many researchers rely on iPSCs from a limited pool of donors, which overlooks inter-individual variability in disease manifestation and drug response. By generating organoids from a wider variety of iPSC sources, including those tailored to specific patients and reflecting diverse populations, one can enhance the accuracy of preclinical results. This approach would also pave the way for more personalized treatment options, ultimately leading to better patient outcomes. Brain organoids exhibit significant potential for modeling rare neurological and neurodevelopmental disorders yet are underutilized. While animal models often struggle to replicate the subtle characteristics of these conditions, organoids generated from patient iPSCs offer a distinct opportunity to investigate disease mechanisms and evaluate potential drug candidates in a relevant cellular environment. Establishing brain organoid biobanks, comprising organized collections of patient-derived and genetically modified organoids, will be essential for facilitating large-scale, disease-oriented research initiatives.

Despite their potential, brain organoids face substantial challenges regarding standardization [94]. Variability in differentiation protocols, culture conditions, and batch-to-batch reproducibility impedes their widespread adoption. Therefore, establishing standardized protocols and robust quality control measures is imperative to ensure consistent organoid generation, maturation, and phenotypic stability across research laboratories. Another essential consideration is integrating the microenvironment with the ECM. Current organoid models lack a comprehensive array of brain-resident and supportive cell types, including vascular, immune, and glial cells. Incorporating these components through co-culture systems or bioengineering techniques can enhance physiological relevance, particularly in modeling diseases characterized by complex cell–cell interactions.

The use of brain organoids in studying immunoneurological disorders is an exciting application [249]. Conditions like multiple sclerosis and autoimmune encephalitis involve complex interactions between the immune system and the nervous system, which are difficult to replicate with conventional models. Progress in incorporating microglia, peripheral immune cells, and cytokine environments into organoids and assembloids could lead to more accurate representations of neuroinflammation and neuroimmune signaling [250]. Additionally, organoid models are starting to explore neuropsychiatric disorders like addiction [251], schizophrenia [252], and bipolar disorder [253], which have traditionally been difficult to model using conventional methods. While the complex nature and circuit-level dysfunction of these conditions pose challenges for modeling, organoids could aid in revealing intrinsic cellular factors and act as platforms for testing new psychoactive or neuroprotective substances [253]. Lastly, the ability to utilize brain organoids to model lysosomal storage diseases and other genetic metabolic disorders [254] paves the way for understanding mechanisms and testing therapies. These conditions typically encompass intricate pathways of multicellular degeneration, which align well with the multicellular structure of organoid systems.

## 12. Conclusions

Advances in stem cell biology, CRISPR technology, and the development of complex brain organoids and assembloids, combined with advanced analysis technologies such as omics approaches and electrophysiological microelectrode arrays, are expanding our understanding of the disease mechanisms underlying a vast array of neurological and neuropsychiatric disorders. It can be expected that over the next few years, the integration of technological advances in the generation of more homogenous brain organoids with screening robotics and the expanded development of accurate disease models for a wide array of brain disorders will advance the development of drugs for neurological disorders, neuropsychiatric diseases, substance abuse disorders, rare brain diseases, and other brain disorders previously thought impossible to treat.

## Figures and Tables

**Table 1:** Brain organoid disease models.

Organoid Type	Disease Modeled	Aim/Methods	Main Findings	Ref.
GBM brain organoid	Glioblastoma	HiPSCs were mutated with genes from the proneural and mesenchymal subtypes.	Mutations linked to GBM disrupted neural development within brain organoids.	[135]
GBM brain organoid	Glioblastoma	Patient-derived cells.	Organoids maintained characteristics of parental GBM.	[136]
GBM brain organoid	Diffuse midline glioma (DMG)	Produced a neuroimmune-competent brain organoid by fusing microglia organoids with DMG organoids.	Replicated DMG infiltration pattern, with microglia mobility and interaction with tumor cells influenced by external factors and microenvironment.	[137]
Cerebellar	Medulloblastoma (MB)	Produced an MB organoid by the CRISPR/Cas9 gene editing of the PTCH1 gene.	Loss of PTCH1 was associated with disrupted neuronal growth and MB.	[138]
Cerebellar	Medulloblastoma (MB)	Generated an SHH (Sonic Hedgehog)-MB cell line organoid model by co-culturing SHH-MB lines with nonmalignant cerebellar organoids to replicate the tumor microenvironment (TME).	SHH-MB cells in cerebellar organoid co-culture mimicked in vivo malignant states, with organoid microenvironment regulating SHH-MB cell states.	[139]
Cerebellar	Medulloblastoma (MB)	Modeled Group 3 medulloblastoma by overexpressing Otx2 and c-Myc genes.	Organoids exhibited DNA methylation like Group 3 MB, and treatment with Tazemetostat diminished Otx2/c-MYC tumorigenesis.	[140]
Cerebral	Autism Spectrum Disorder (ASD)	Identified the FABP7/MEK axis as a potential drug target in idiopathic ASD using ASD patient-derived iPSCs.	ASD organoids showed premature differentiation with impaired expression of FABP7.	[141]
Cerebral	ASD	Studied the effects of the elimination of the CNTNAP2 gene in ASD, using organoids generated from CRISPR-edited iPSCs.	CNTNAP2 plays vital role in development of brain during early stages and may be linked to mechanisms underlying ASD.	[142]
Cerebral	ASD	Investigated the impact of an underdeveloped choroid plexus on the onset of ASD, using ASD patient-derived iPSCs.	Undeveloped choroid plexus is prevalent characteristic in ASD pathology.	[143]
Cerebral	ASD	Investigated the relationship between EGR1 and SHANK3, using cerebral organoids at different developmental stages.	EGR1 expression influenced SHANK3 transcription.	[144]
Cerebral	ASD	Explored the role of microglial phagocytosis in synapses, using a microglia-SCN2A-mutated cerebral organoid.	Role of microglia in eliminating surplus synapses is evolutionarily conserved.	[145]
Forebrain	ASD	Analyzed alterations in the PTEN gene, using CRISPR-edited iPSCs.	PTEN regulated several crucial neurodevelopmental processes in early and late stages of development.	[146]
Cortical	ASD	Generated hESCs with CHD8 haploinsufficiency and CHD8 mutations and used these cells to recapitulate a macrocephaly-like phenotype in cerebral organoids.	CHD8 mutations affected proliferation and differentiation dynamics with accelerated and delayed generation of inhibitory and excitatory neurons, respectively. CHD8 haploinsufficiency also disrupted neurodevelopmental trajectories.	[147]
Cortical	ASD	Explored the genetic etiology of ASD, using organoids derived from sons with idiopathic ASD and unaffected fathers.	Macrocephalic and normocephalic probands showed different expression of transcription factors during early cortical development.	[148]
Cortical	ASD	Analyzed the alteration of SYNGAP1, using patient-derived organoids.	SYNGAP1 haploinsufficiency led to abnormal cytoskeleton dynamics, disrupting and dividing orientation of radial cells.	[149]
Cortical	Epilepsy (Focal cortical dysplasia II)	Investigated the role of GATOR1 subunit NPRL3, using iPSCs derived from an FCD II patient.	Produced novel organoid model to investigate NPRL3-related epilepsy. Findings aligned with hyperactivation of mTOR pathway observed in FCD.	[150]
Cortical	Epilepsy (PCDH19-clustering epilepsy)	Studied the role of PCDH19, using organoids produced from CRISPR-edited female iPSCs.	PCDH19 is crucial for organizing radial glial cells and early cortical development in human ventricular zone.	[151]
Cerebral	Epilepsy (GLUT1-Deficiency syndrome; GLUT1-DS)	Investigated the role of the GLUT1 gene, using patient-derived organoids.	GLUT1-DS organoids showed lower cell density and smaller dimensions, exhibiting epileptiform activity and increased glucose sensitivity deprivation.	[152]
Cerebral	Epilepsy (Tuberous sclerosis; TSC)	Demonstrated how human-specific developmental processes contribute to malformations of cortical development, utilizing patient-derived iPSCs with TSC2 mutations.	Identified caudal late interneuron progenitor (CLIP) cells in TSC organoids that exhibit excessive proliferation, resulting in overabundance of interneurons, brain tumors, and cortical malformations.	[153]
Cerebral	Cerebral Palsy	Analyzed the effects of the loss of the TYW1 gene, using organoids produced from CRISPR-edited iPSCs.	Loss of TYW1 led to impaired differentiation of neurons.	[154]
Cerebral	Cerebral palsy	Evaluated the effectiveness of minocycline on neonatal hypoxic insult (NHI) using an organoid model of hypoxia.	Minocycline reduced impact of NHI on organoids.	[155]
Telencephalic	ADHD	Explored the emergence of ADHD in the early cerebral cortex through patient-derived organoids.	In ADHD organoids, cortical maturation was delayed, along with alterations in qualities of neural stem cells and development of layered structures.	[156]
Assembloids (Cortico-striatal)		Developed a human in vitro model that recapitulates the dopaminergic innervation of the striatum and cortex.	Assembloids comprised ventral midbrain–striatum–cortical organoids, which can be used to study dopaminergic neuron maturation, innervation, and functions.	[157]
Assembloids (Striato-nigral)		Generated Str-SN assembloids by fusing striatum-like and midbrain SN-like organoids.	Model showed significant medium spiny neuron (MSN) projections from striatum to SN.	[158]
Assembloids (Cortico-motor)		Developed cortico-motor assembloids by fusing cerebral/hindbrain/spinal and skeletal muscle organoids.	Model demonstrated that corticofugal neurons project and establish connections with spinal organoids. Optogenetic activation of cortical spheroids elicited significant contractions in 3D muscle.	[159]
Assembloids (Forebrain)		Combined CRISPR screening with assembloids to explore 425 neurodevelopmental disorder (NDD) genes related to interneuron development.	Discovered 33 candidate genes from interneuron migration screen using more than 1000 forebrain assembloids. Emphasized efficacy of CRISPR-assembloid platform in studying NDDs and human development.	[160]
Assembloids (Neuroimmune)		Created neuroimmune assembloids using cortical organoids and microglia.	Model serves as tool for exploring interactions between neural and immune cells.	[161]
Assembloids (Neuron–astrocyte)		Developed a model that more accurately mimics the aging brain and Parkinson’s disease (PD)-related pathologies by incorporating astrocytes.	Neuron–astrocyte assembloids highlighted role of glial cells in neurodegeneration processes.	[162]
Assembloids (Thalamocortical)		Created thalamocortical assembloids by fusing thalamus and cortical organoids.	scRNA analysis showed that over 80% of thalamic organoid cells were glutamatergic neurons. Assembloids formed reciprocal projections and synapses, demonstrating short-term plasticity. Valuable model for studying synaptic plasticity in human neural system circuits.	[163]
Assembloids (Thalamocortical)		Investigated the contribution of genetic variants in T-type calcium channels in the early development of the human thalamocortical pathway.	M15331V CACNA1G variant led to changes in T-type currents in thalamic neurons, which resulted in hyperactivity of thalamic and cortical neurons and abnormal thalamocortical connectivity.	[164]
Assembloids (Cortical–blood vessel)		Developed assembloids by merging cortical and blood vessel organoids.	Observed notable increase in microglia and astrocytes in brain organoids, providing platform to study neurotrophic diseases such as COVID-19 and its correlation with AD.	[165]
Assembloids (Somatosensory)		Developed human ascending somatosensory assembloids (hASAs).	Four-part assembloids integrating somatosensory, spinal, thalamic, and cortical organoids model spinothalamic pathway. Transcriptomic profiling confirmed key cell types, and chemical stimulation showed coordinated response.	[166]

**Table 2:** Brain organoids in drug discovery.

Organoid	Disease	Aim	Strategy	Main Findings	Ref.
Midbrain		Created a scalable and automated platform for generating and analyzing organoids.	Automated high-throughput workflow, high-content imaging (HCI).	Organoids with minimal variability in size, morphology, and cellular composition within and across batches. RNA sequencing and HCI confirmed reproducibility.	[69]
Cerebral	Alzheimer’s disease (AD)	Developed a drug screening model for AD and evaluated FDA-approved drugs that can penetrate the blood–brain barrier.	Mathematical modeling, high-content screening.	Generated a network-based computational model of AD and identified FDA-approved compounds that alleviate AD-related phenotypes.	[240]
Cortical		Aimed to establish a scalable and automated platform for high-throughput screening (HTS) using iPSC-derived brain organoids.	High-content imaging (HCI), multi-electrode arrays (MEAs), and single cell-calcium imaging.	Demonstrated the feasibility of using iPSC-derived brain organoids in an automated HTS platform, offering a promising tool for drug discovery and neurotoxicity.	[243]
Cerebral	Developmental neurotoxicity (DNT)	Aimed to enhance the evaluation of DNT by employing a dynamic culture system.	Pillar/perfusion plate platform.	A dynamic culture approach offers a high-throughput platform for assessing the DNT of various compounds.	[244]
Cerebral		Aimed to develop a simple and reproducible method to enhance scalability and constituency in organoid production.	Pillar plate/ultra-low attachment well plate.	Uniform cerebral organoids offer a scalable and efficient platform for high-throughput organoid-based assay.	[245]
Cerebral	Glioma	Described a method that reproducibly generates thousands of organoids across multiple iPSC lines.	High-quantity (Hi-Q) brain organoid approach.	Enabled a medium-throughput drug screen that identified selumetinib and fulvestrant as inhibitors of glioma invasion.	[246]
Patient-derived organoids (PDO)	Melanoma brain metastases	Created patient-derived organoids from melanoma brain metastases (MBM-PDOs) and evaluated the practicality of utilizing them as a model for testing targeted therapy drugs in vitro.	Cell viability assessment after MBM-PDOs were exposed to drugs.	Utilizing FDA-approved inhibitors for BRAF and MEK, demonstrated the practicality of employing MBM-PDOs for identifying targeted therapeutics.	[247]

## Data Availability

No new data were created or analyzed in this study.
